# miRNA‐encapsulated abiotic materials and biovectors for cutaneous and oral wound healing: Biogenesis, mechanisms, and delivery nanocarriers

**DOI:** 10.1002/btm2.10343

**Published:** 2022-06-28

**Authors:** Asmita Deka Dey, Satar Yousefiasl, Arun Kumar, Farnaz Dabbagh Moghaddam, Ilnaz Rahimmanesh, Mohamadmahdi Samandari, Sumit Jamwal, Aziz Maleki, Abbas Mohammadi, Navid Rabiee, Ana Cláudia Paiva‐Santos, Ali Tamayol, Esmaeel Sharifi, Pooyan Makvandi

**Affiliations:** ^1^ Chitkara College of Pharmacy Chitkara University Punjab India; ^2^ School of Dentistry Hamadan University of Medical Sciences Hamadan Iran; ^3^ Department of Biology, Science and Research Branch Islamic Azad University Tehran Iran; ^4^ Institute for Photonics and Nanotechnologies, National Research Council, Via Fosso del Cavaliere, 100 Rome Italy; ^5^ Applied Physiology Research Center Cardiovascular Research Institute, Isfahan University of Medical Sciences Isfahan Iran; ^6^ Department of Biomedical Engineering University of Connecticut Farmington Connecticut USA; ^7^ Department of Psychiatry, Yale School of Medicine Yale University New Haven Connecticut USA; ^8^ Department of Pharmaceutical Nanotechnology, School of Pharmacy Zanjan University of Medical Sciences Zanjan Iran; ^9^ Zanjan Pharmaceutical Nanotechnology Research Center (ZPNRC) Zanjan University of Medical Sciences Zanjan Iran; ^10^ Cancer Research Centre Shahid Beheshti University of Medical Sciences Tehran Iran; ^11^ Department of Chemistry University of Isfahan Isfahan Iran; ^12^ Department of Physics Sharif University of Technology Tehran Iran; ^13^ School of Engineering Macquarie University Sydney New South Wales Australia; ^14^ Department of Pharmaceutical Technology Faculty of Pharmacy of the University of Coimbra, University of Coimbra Coimbra Portugal; ^15^ LAQV, REQUIMTE, Department of Pharmaceutical Technology Faculty of Pharmacy of the University of Coimbra, University of Coimbra Coimbra Portugal; ^16^ Department of Tissue Engineering and Biomaterials, School of Advanced Medical Sciences and Technologies Hamadan University of Medical Sciences Hamadan Iran; ^17^ Istituto Italiano di Tecnologia, Centre for Materials Interfaces Pontedera Italy; ^18^ School of Chemistry, Damghan University Damghan Iran

**Keywords:** abiotic nanomaterials, chronic wounds, miRNA delivery, nanobiovectors, oral mucosa wound, viral and nonviral nanocarriers

## Abstract

MicroRNAs (miRNAs) as therapeutic agents have attracted increasing interest in the past decade owing to their significant effectiveness in treating a wide array of ailments. These polymerases II‐derived noncoding RNAs act through post‐transcriptional controlling of different proteins and their allied pathways. Like other areas of medicine, researchers have utilized miRNAs for managing acute and chronic wounds. The increase in the number of patients suffering from either under‐healing or over‐healing wound demonstrates the limited efficacy of the current wound healing strategies and dictates the demands for simpler approaches with greater efficacy. Various miRNA can be designed to induce pathway beneficial for wound healing. However, the proper design of miRNA and its delivery system for wound healing applications are still challenging due to their limited stability and intracellular delivery. Therefore, new miRNAs are required to be identified and their delivery strategy needs to be optimized. In this review, we discuss the diverse roles of miRNAs in various stages of wound healing and provide an insight on the most recent findings in the nanotechnology and biomaterials field, which might offer opportunities for the development of new strategies for this chronic condition. We also highlight the advances in biomaterials and delivery systems, emphasizing their challenges and resolutions for miRNA‐based wound healing. We further review various biovectors (e.g., adenovirus and lentivirus) and abiotic materials such as organic and inorganic nanomaterials, along with dendrimers and scaffolds, as the delivery systems for miRNA‐based wound healing. Finally, challenges and opportunities for translation of miRNA‐based strategies into clinical applications are discussed.

## 1 INTRODUCTION

Chronic wounds, particularly venous ulcers, diabetic ulcers, arterial insufficiency ulcers, and pressure ulcers, are arduous and exorbitant to cure.^1^ Globally, many people suffer from chronic wounds with a growing prevalence every year. The Considerable but often unrecognized impact on patients and the healthcare system have turned chronic wounds into a silent epidemic. The increase in chronic wounds can be attributed to various factors such as the aging population and the associated rise in comorbidities and lifestyle disorders, including diabetes, obesity, chronic venous hypertension, and peripheral artery diseases.^2^, ^3^ Chronic wounds are not only a substantial health issue, but they also have substantial monetary and psychological consequences. According to the presented reports, approximately 6.5 million of the US population are agonized by non‐healing, chronic wounds causing hefty monetary distress of 25 billion dollars per annum.^4^ The burden of these medical morbidities is vast, thereby driving a substantial interest in novel medicines with better clinical efficacy.

In a normal physiological condition, the cascade of hemostasis, inflammation, proliferation, and remodeling leads to tissue regeneration and wound healing.^5^, ^6^ However, if the cascade is interrupted, wound healing will be impaired. If the wound does not heal for more than 90 days, it is referred to as chronic wound.^7^ Chronic wounds are typically represented by tissue hypoxia, excessive inflammation, and persistent bacterial infections and biofilms.^8^, ^9^, ^10^ To treat chronic wounds, an array of strategies including biological factor and cell therapies and electromechanical stimulation of the injury site have been explored.^11^ The harsh environment of chronic wounds has limited the success of exogenous biological factor therapies due to the instability of large proteins and their limited distribution in the healing tissue.^12^

miRNA, a small noncoding endogenic RNA, has an average length of 22 nucleotide molecules and regulates the post‐transcriptional gene expression.^13^ Various miRNAs are being established for tissue regeneration upon injury and promising results are being reported.^14^, ^15^ For example, miRNA‐155, miRNA‐21, miRNA‐130a, miRNA‐31, miRNA‐132, miRNA‐378a, and miRNA‐198 are the predominant miRNAs demonstrating various roles in the process of wound healing.^16^, ^17^ Among several miRNAs known to contribute in tissue regeneration, miRNA‐21 is demonstrated to perform two vital roles in the wound healing management, primarily by modulating the inflammatory and by modulating the proliferative phases, the two critical physiological healing stages needed for enhanced healing and reduction of scar formation.^18^, ^19^ In rat models with diabetic wounds, miR‐27b and miR‐15b were witnessed to play a significant part in the process of angiogenesis together with accelerating the closure of wounds.

The promise of miRNAs in healing skin wounds attracted increasing attention toward their expanded applicants for a wide array of modern therapeutic approaches in wound healing.^20^ For instance, miRNA‐126 is engaged in angiogenesis, one of the most crucial factors in wound healing process. Upon loading the miRNA‐126 on liposomes modified using polyethylene glycol and delivering it to ischemic wounds, the angiogenic factor, vascular endothelial growth factor (VEGF), was triggered. The systemic circulation was enhanced, aided to fuel angiogenesis, and ultimately promoted wound healing.^21^ However, an efficient delivery is challenging in miRNA therapy due to their short‐term stability and limited cellular permeability, which is why various biovectors like adenovirus, lentivirus, and abiotic nanomaterials such as gold and silver nanoparticles (NPs) have been used to surpass the clinical limitations.^22^

The current review explores miRNA synthesis, structure, functionality, and its contribution in different stages of wound repair in dermal and oral tissues. The review outlines various mechanisms of miRNA action, its therapeutic capability for oral mucosa, and dermal tissue wound healing. In addition, the delivery platforms including biovectors and nonviral vectors materials (e.g., nanoscale particles, dendrimers, and hydrogels) have been presented. Besides, the current article highlights the benefits of miRNA therapy and thus scrutinizes the success of this versatile therapeutic entity as a developing approach toward chronic wound healing.

## 2 BIOGENY OF miRNA

The genetic makeup of human beings comprises an excess of over 500 miRNAs, and each of them can carry out the suppression of hundreds of genes.^23^ miRNAs in animals are strongly associated with almost every pathophysiological process conducive to development as they are proposed to target over 50% of the protein‐coding transcript.^24^ Their functionalities add to numerous disorders, including malignant growths, cardiovascular diseases, and neurodegenerative disorders, so miRNAs are generally utilized as biomarkers and therapeutics in medicine.^25^, ^26^, ^27^

Small RNAs in eukaryotes can suppress genetic material and unwanted cell transcripts.^28^, ^29^ Also, small RNAs are usually between 20 and 30 nucleotides in length and are associated with argonaute family proteins.^30^ These proteins are divided into miRNA, siRNA, and piwi (an endoribonuclease domain)‐interacting RNA.^30^ In examining the structure, miRNAs are usually a dominant class of small RNAs consisting of approximately 22 nucleotides and are produced by Dicer, two RNase III proteins, and Drosha.^31^ Transcription, processing by Drosha and Dicer, loading onto argonaute family proteins, and turnover are steps during which miRNA is regulated.^32^, ^33^

RNA polymerase II/III transcribes miRNA genes, and the primary transcript has a hairpin structure where miRNA sequences are embedded.^34^ Succeeding transmission, the miRNA undergoes the initial cleavage step where the miRNA is cleaved into primary‐miRNA (a stem of 33–35 bp, a terminal loop and single‐stranded RNA segments at both the 5′ and 3′ sides) through RNA polymerase II/III.^34^ The p53, MYC, zinc finger E‐box‐binding homeobox 1 and zinc finger E‐box‐binding homeobox 2, and myoblast determination protein 1 (as transcription factors) positively or negatively regulate miRNA expression.^35^ In the next step, Drosha is cropping the stem‐loop to release a small hairpin‐shaped RNA that is made up of about 65 nucleotides and is called precursor miRNA.^36^ Drosha, a nonspecific double‐stranded RNA, inserts staggered cuts in every strand of RNA‐helix and with DiGeorge syndrome critical region 8 (DGCR8), also known as pasha in *drosophila melanogaster* and as pash‐1 in *caenorhabditis elegans* forms a complex called microprocessor.^37^, ^38^ DGCR8, a vital cofactor for Drosha, comprises two dual‐stranded RNA that codes for binding domains and aids in the formation of protein complexes.^39^ The interaction of Drosha with DGCR8 takes place in the nucleus.^40^

Human recombinant Drosha solely demonstrates nonspecific RNase activity; however, the addition of DGCR8 makes it specific for processing precursor miRNA.^41^ Precursor miRNA are preserved in the form of internal loops and bulges that frequently emerge in the definite positions of the miRNA stem.^42^ This allows proper enzyme‐catalyzed processing, thus resulting in miRNA maturation.^42^ The consequential precursor miRNA is migrated to the cytoplasm where maturation can be completed. The protein exportin‐5 forms a transport complex with Guanosine triphosphate‐binding nuclear protein Ran‐GTP and a precursor miRNA.^43^, ^44^ The reduction of Guanosine triphosphate impairs the migration of precursor miRNA‐bound Ran. Therefore, it is considered that exportin‐5 depends on the nuclear Ran‐GTP to perform its functions.^45^

Upon cleaving by an RNase III enzyme known as Dicer, precursor miRNA is converted into 18–24 nucleotide double‐stranded RNA.^46^ After maturation, this cleaves precursor miRNA, thus turning it into a miRNA duplex.^46^ The enzyme Dicer works via intramolecular dimerization of its two domains, the double‐stranded RNA binding domain and the flanked RNA binding domain, that is, Piwi/Ago/Zwille.^47^ After cleavage, the resulting miRNA duplex calls for the release of any one of the strands to receive entry into argonaute for functions.^48^ The majority of the miRNAs are encapsulated in the RNA‐induced silencing complex to form miRNA‐containing RNA‐induced silencing complex.^49^ RNA‐induced silencing complex assembly includes loading RNA duplex and its unwinding steps.^50^

**FIGURE 1:**
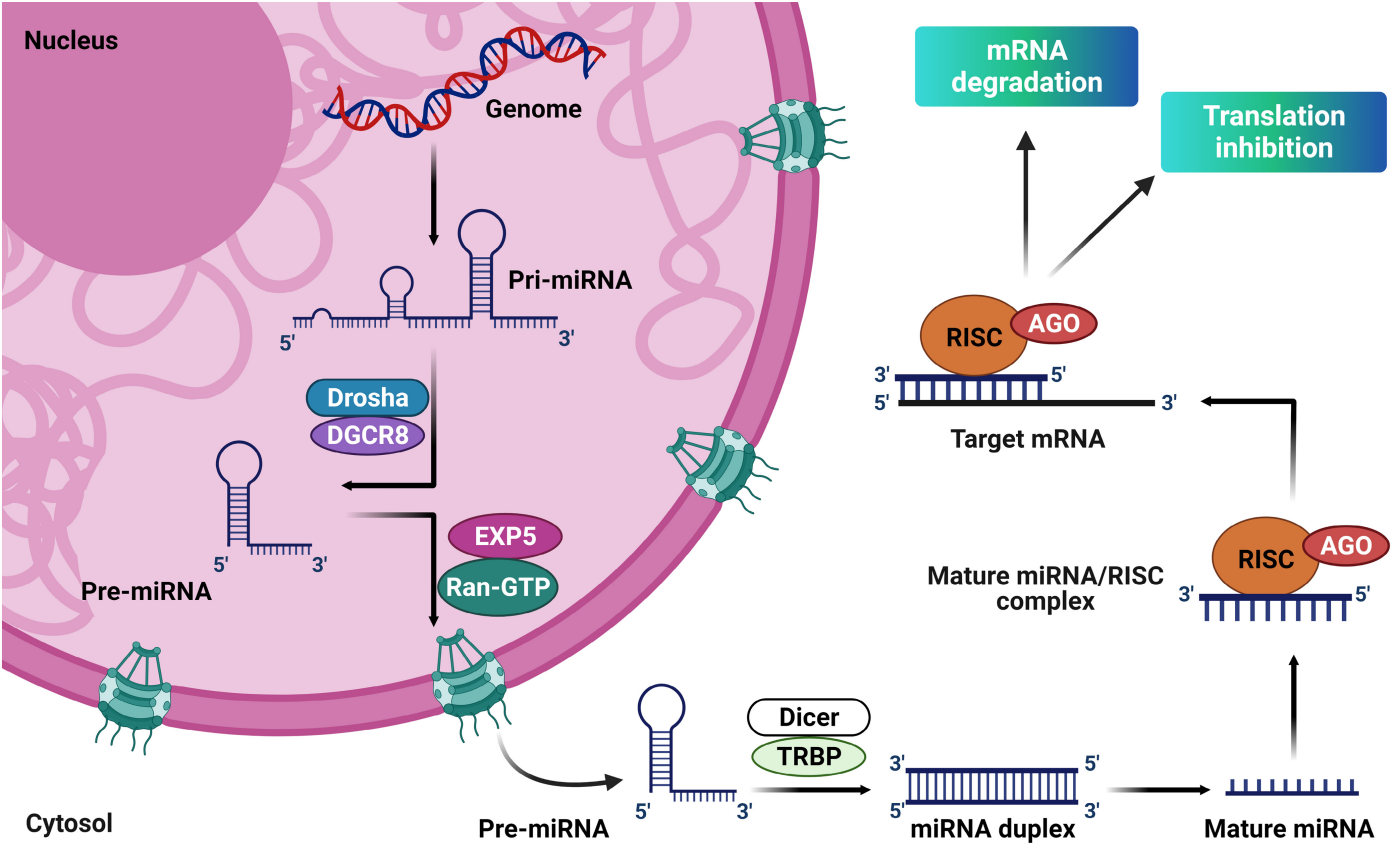
Graphic representation of the biogenesis of miRNA. RNA polymerase II/III carries out miRNA transcription from genomic DNA to construct the pri‐miRNA in the nucleus. Drosha and its cofactor DGCR8 then cleave the pri‐miRNA to form Pre‐miRNA. Exportin‐5 and Ran‐GTP transports the pre‐miRNA into the cytoplasm. In the cytoplasm, the Dicer and TRBP recognize pre‐miRNA and cut into dsRNA which matures and thus resulting in a miRNA duplex. Matured miRNA duplex is loaded in the RISC to form the mature miRNA molecules. AGO, argonaute; DGCR8, DiGeorge syndrome critical region 8; Pre‐miRNA, precursor miRNA; Pri‐miRNA, primary miRNA; RISC, RNA‐induced silencing complex; TRBP, TAR RNA binding protein

In the miRNA duplex, the strand fated to transform into the matured miRNA is referred to as the guide strand, while the remaining second strand of the duplex is termed star or passenger strand.^48^ After cleavage, miRNA duplex needs to release the passenger strand to receive entry into argonaute to function.^48^ The guide strand of the miRNA duplex is encapsulated and subsequently directs the enzyme to form a miRNA‐containing RNA‐induced silencing complex.^49^ After binding to the mRNA, argonaute recruits the trinucleotide repeat containing six proteins, a scaffold protein tethering effector protein to destabilize and translationally repress target mRNAs by inducing their decapping and deadenylation (Figure 1).

## 3 ROLE OF miRNA IN WOUND HEALING PHASES AND IMMUNOGENICITY

The wound healing process consists of complex and dynamic physiological pathways that promptly occur to hemotasis and inflammation, proliferation, and regeneration within the wound and tissue.^8^, ^51^, ^52^ Various miRNAs have been found to date to undergo either downregulation or upregulation during four phases of wound healing. Some of the essential miRNAs having a significant involvement in wound repair and immunogenicity pathways are mentioned in Table 1.

**TABLE 1:** miRNAs in different stages of wound healing and their target of action

	miRNA	Expression level in wound healing	Target	Ref
Pro‐inflammatory	miRNA‐155	Upregulated	Suppressor of cytokine signaling 1, Src homology 2 domain containing inositol polyphosphate 5‐phosphatase 1, IL‐12	53
	miRNA‐140	Overexpression	Platelet‐derived growth factor receptors	54
Anti‐inflammatory	miRNA‐203	Upregulated	IL‐24, tumor necrosis factor‐α	55
	miRNA‐16	Overexpression	Endoperoxide synthase 2	56
	miRNA‐21	Upregulated	Programmed cell death protein, Phosphatase and tensin homolog	57
	miRNA‐105	Overexpression	Toll‐like receptor 2	58
	miRNA‐125b	Overexpression	Tumor necrosis factor‐α	59
	miRNA‐146a, b	Upregulated	Signal transducer and activator of transcription 1, tumor necrosis factor receptor associated factor 6, interleukin‐1 receptor‐associated kinase 1, tumor necrosis factor‐α, endoperoxide synthase 2	60
	miRNA‐223	Downregulated	Myocyte‐specific enhancer factor 2C	61
Proliferation	miRNA‐155	Upregulated	Fibroblast growth factor 7, keratinocyte growth factor	62
	miRNA‐184	Upregulated	Protein kinase B	63
	miRNA‐198	Overexpression	Laminin subunit gamma‐2, diaphanous related formin 1, plasminogen activator, urokinase	64
	miRNA‐203	Downregulated	Ras association (RalGDS/AF‐6), Ras‐related nuclear protein	65
	miRNA‐483‐3p	Overexpression	Yes1 associated transcriptional regulator, mitogen‐activated protein kinase‐activated protein kinase‐2, Marker of proliferation Ki‐67	66
	miRNA‐21 miRNA‐31	Upregulated Upregulated	TIAM rac1 associated GEF 1, TIMP metallopeptidase inhibitor 3 Epithelial membrane protein	67
	miRNA‐210	Overexpression	Iron–sulfur cluster assembly enzyme, E2F transcription factor 3	68
	miRNA‐99	Downregulated	AKT serine/threonine kinase 1, Insulin like growth factor 1 receptor, mechanistic target of rapamycin kinase	69
	miRNA‐205	Overexpression	Rho associated coiled‐coil containing protein kinase 1, SH2‐containing phosphatidylinositol 3,4,5‐trisphosphate 5‐phosphatase	70
Pro‐angiogenic	miRNA‐17‐92	Overexpression	Thrombospondin 1, connective tissue growth factor	71
	miRNA‐130a	Downregulated	Homeobox A5, growth arrest homeobox	72
	miRNA‐296	Upregulated	Hepatocyte growth factor‐regulated tyrosine kinase substrate	73
Anti‐angiogenic	miRNA‐92a	Overexpression	Integrin‐α5	74
	miRNA‐15b	Upregulated	VEGF	75
	miRNA‐17		Janus kinase 1	76
	miRNA‐503	Downregulation	Cell division cycle 25A, Cyclin E1	77
Remodeling	miRNA‐29b miRNA‐29c miRNA‐192/215	Downregulated Downregulated Downregulated	Smads, β‐catenin TAB‐1, collagen I and II Interacting protein 1	78 79 80

### 3.1 First phase: miRNA in the inflammatory stage

**FIGURE 2:**
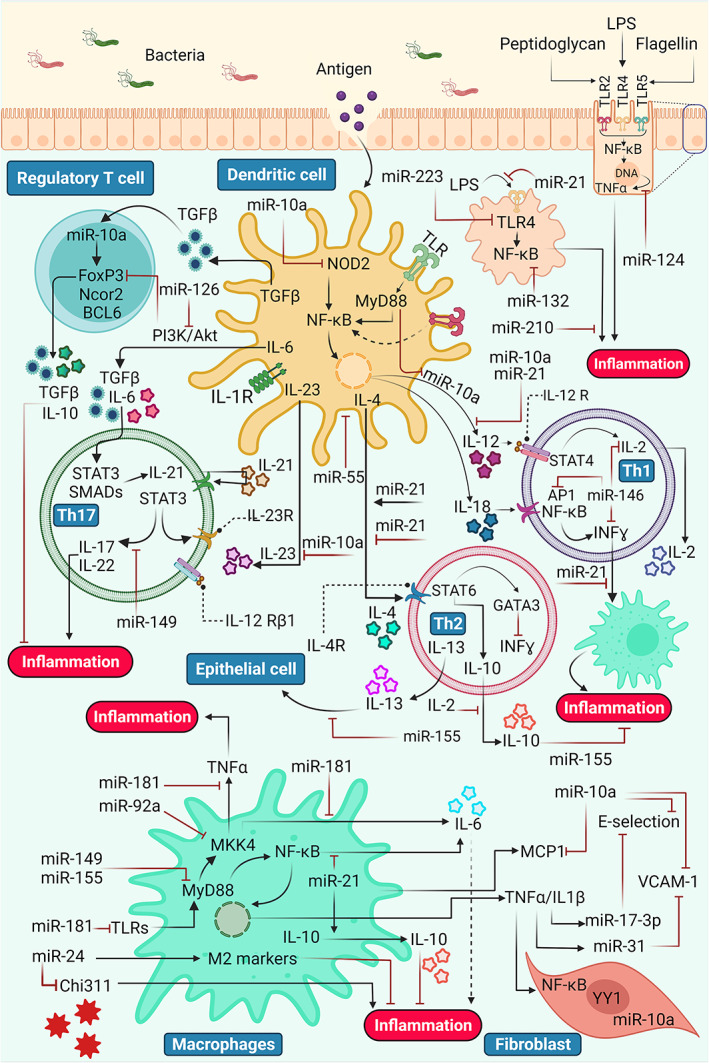
Targeted pathways of some anti‐inflammatory miRNAs are observed to control and regulate the inflammatory response. In addition to activating some of the mechanisms involved in this process, various miRNAs are involved in inflammatory processes and inflammatory diseases by creating significant loops of negative feedback.^80^ AP1, activator protein 1; GATA3, GATA binding protein 3; IFNγ, interferon gamma; IL, interleukin; LPS, lipopolysaccharide; MCP1, monocyte chemoattractant protein‐1; NF‐kB, nuclear factor kappa‐light‐chain‐enhancer of activated B cells; NOD2, nucleotide‐binding oligomerization domain‐containing protein 2; STAT3, signal transducer and activator of transcription 3; STAT6, signal transducer and activator of transcription 6; Th1, T helper type1; Th17, T helper type17; Th2, T helper type2; TLR, Toll‐like receptor; TNFα, tumor necrosis factor alpha; VCAM‐1, vascular cell adhesion molecule 1

Inflammation is known as a biological and pathophysiological reaction complex caused by tissue damage or infection. Molecular networks play a role in the functioning of regulatory pathways. In addition to protein regulators, miRNAs appear to be significant regulators of inflammation and modulate the onset and end of inflammation by suppressing or amplifying signaling (Figure 2).^81^ Inflammation is the primary stage of wound healing and commences immediately following the disruption of the stratum corneum and activates the cascade of events associated with the clotting process.^82^ Since hemostasis takes place via the development of fibrin clots, immune cells discharge the chemokines and cytokines like platelet‐derived growth factor, platelet factor‐IV, transforming growth factor‐β, and tumor necrosis factor‐α (TNF‐α) into the wounding area.^83^, ^84^ Immune cells like monocytes and neutrophils are then passively released into the wound via the injured blood vessels.^85^ The effused monocytes develop into macrophages and perform phagocytic actions. Monocytes might be pro‐angiogenic and can be either pro‐inflammatory or anti‐inflammatory.^86^

Furthermore, due to infringement of the protective dermal layer, the underlying tissues get exposed to the pathogens, and the chance of infections increases manifold.^87^ The released neutrophils then arrive at the wound site via the chemokine signaling and cytokine mentioned above and elicit their role of cleansing and killing the invasive microorganisms.^88^ This inflammatory signaling and the expression of receptor genes are several ways in which miRNA regulation of the inflammatory stage is observed. Various miRNA was recognized at this phase to be fundamental. For instance, miRNA‐146a negatively regulates the inflammatory stage responses associated with the intact skin.^89^ The miRNA‐146a expression is enhanced in the epidermal keratinocytes prompted by the toll‐like receptors 2, 3, 4, and 5.

Moreover, the negative regulation suggests that miRNA‐146a might promote the resolution of inflammation.^90^ A significant downregulation in the appearance of miRNA‐146a was witnessed in diabetic wounds of mice in contrast to the nondiabetic models.^91^ Furthermore, investigations suggested that this miRNA also targets and silences the pro‐inflammatory mediators like interleukin‐1 receptor‐associated kinase one and TNF receptor‐associated factor 6.^92^ miRNA 155 is another essential miRNA for the immune cell. Both miRNA‐155 and miRNA‐146a regulate macrophages, which further stimulate the generation of cytokines and the growth factors required for the differentiation of monocytes to macrophages.^93^ Moreover, upregulation of miRNA‐155 in the inflammatory phase has been audited in mouse models, demonstrating that miRNA‐155‐specific inhibitor therapy can efficaciously mitigate the inflammatory cellular proliferation at the wound area and, therefore, ameliorate the construction of restored tissues.^51^, ^94^, ^95^ miRNA‐132 has an anti‐inflammatory role in this phase and limits the excessive production of pro‐inflammatory cytokines.^96^, ^97^ Inflammation has stimulated the miRNA‐132 expression in the leukocytes^81^ and a change in its level of expression in the course of transformation from the inflammation phase to the second stage of healing that is proliferation.^97^

miRNA mimics are double‐stranded RNA molecules generated to resemble mature miRNA duplexes. Chemical alterations and nucleotide changes in the passenger strands are typically given to miRNA mimics in order to improve their stability, promote guide miRNA loading to RISC, and selectively exclude the passenger strand.^98^ The topical administration of miRNA‐132 mimics loaded liposomes at the wound area resulted in a rapid recovery of the dermal wound in diabetic mice and re‐epithelization of the human ex vivo dermal wounds through the suppression of the pro‐inflammatory cytokines in keratinocytes and macrophages. Furthermore, miRNA‐132 also improves the changeover from inflammation to the proliferation stage during the healing process via targeting the heparin‐binding endothelial growth factor.^99^ Additionally, miRNA‐21 is also evidenced to be crucial in the resolution of inflammation.^100^ Furthermore, miRNAs including miRNA‐191, miRNA‐200, and several others are intricate in regulating inflammation in wound healing.

Although miRNAs have an essential role in promoting and inducing inflammation, they also downregulate or end the stage.^93^ Protraction of this stage leads to damaged tissues and impedes proper wound healing, resulting in chronic wounds.^94^ It was confirmed that the suppression of miRNA‐155 yielded a reduction in inflammation.^101^ With the termination of the inflammatory stage by the decreased neutrophils count and macrophages, the proliferation phase is initiated.

By increasing miRNA‐9 expression in the cerebral cortex, factors associated with the NF‐κB signaling pathway such as NF‐κB p65, TNF‐α, and interleukin (IL)‐1β are reduced. Decreased miRNA‐9 is associated with increased synthesis of pro‐inflammatory mediators such as IL‐1β, TNF‐α, IL‐6, and monocyte chemoattractant protein‐1.^102^ On the other hand, miRNA‐173p and miRNA‐31 target E‐selectin and intercellular adhesion molecule 1, respectively. miRNA‐92a reduces inflammation by targeting mitogen‐activated protein kinase 4. miRNA‐99b regulates the expression of inflammatory cytokines IL‐6, IL‐12, and IL‐1β.^63^

The secretion of IL‐6, TNF‐α, chemokine monocyte chemoattractant protein‐1 in human gingival fibroblasts, and IL‐10 increases as the concentration of miRNA‐126 increases.^81^ In addition, miRNA‐126 inhibits inflammation and reactive oxygen species production in human endothelial cells by modulating high mobility group box protein 1 expression.^81^

miR‐142‐3p can regulate murine macrophages synthesis of NF‐κB1, TNF‐α, and IL‐6. miR‐187 expression is induced by IL‐10 (potent anti‐inflammatory cytokine) in Toll‐like receptor 4‐stimulated monocytes.^80^ miRNA‐210 in articular cavities of mice with osteoarthritis was able to exert its anti‐inflammatory effects by inhibiting NF‐κB signaling; also, miRNA‐223 modulated the inflammatory response in human gingival fibroblasts.^80^

### 3.2 Second phase: miRNA in the proliferative stage

The second phase includes epithelialization, angiogenesis, and granulation tissue formation. The proliferative stage of wound healing begins 2–3 days after the abrasion takes place and involves the enrolment of fibroblasts at the injured site to begin granulation tissue formation, collagen deposition, and differentiation of keratinocytes and glycosaminoglycans wound epithelialization, and finally wound closure.^103^, ^104^ Cytokines demonstrate varying actions on the miRNAs present in the fibroblast, including miRNA‐155, which facilitates and enhances the migration of fibroblast.^104^ Fibroblasts are responsible for excreting fibronectin and collagen to build a new extracellular matrix, forming the foundation for granulation tissues.^105^ Additionally, miRNAs viz miRNA‐21, ‐155, ‐99, ‐198, ‐203, ‐205, ‐210, ‐184, and ‐483‐3p functions to govern the construction, differentiation, and transport of keratinocytes.^105^, ^106^, ^107^ The proliferation of keratinocytes is a major activity for achieving wound re‐epithelization, and thus, regulation of ergo miRNA becomes pivotal. During this phase, newer blood vessels are formed to furnish the wound site with abundant oxygen and adequate nutrient supply through angiogenesis or neovascularization. This procedure is important for boosting up the endothelial cell and fibroblast activity. The low oxygen environment induces the hypoxia‐inducible factor to bring about gene activation such as glucose transporter 1 and VEGF to fuel angiogenesis.^103^, ^108^, ^109^ miRNAs sensitive to hypoxia such as miRNA‐23, ‐24, ‐26, ‐27, ‐103, ‐107, ‐181, and ‐213 are under the control of hypoxia‐inducible factor.^110^ Due to this phase, the tissues seem to be erythematous because of the freshly developed network of capillaries. Various miRNAs are engaged in the pro‐angiogenic and anti‐angiogenic regulation such as miRNA‐15b, ‐16, ‐17‐92, ‐126, ‐130a, ‐210, ‐221, ‐222 ‐296, ‐320, ‐378, and ‐503.^111^, ^112^, ^113^, ^114^ Additionally, the keratinocyte migration takes place from the edge to the site of injury and thus initiates proliferation and differentiation to rejuvenate the integrity of the skin. However, several miRNAs may suppress this process, such as miRNA‐198, miRNA‐483‐3p, and miRNA‐203.^115^, ^116^, ^117^

### 3.3 Third phase: miRNA in remodeling stage

The remodeling stage starts after the complete closure of the wound. This phase is a lengthy stage in healable injuries, and the recovery period might range from a few weeks to several months. The remodeling phase involves the extracellular matrix modification, restoration of collagen from type III to type I collagen, and scar formation in the area of provisional granulation tissues.^118^, ^119^ The matrix metalloproteinases and the tissue inhibitors of metalloproteinases have a significant role in matrix remodeling.^119^ miRNA‐335 and miRNA‐129 have shown significant downregulation in diabetic dermal tissues compared to nondiabetic skin tissues.^120^ The straight target for both the miRNAs is transcription factor Sp1 and the overexpression of these miRNAs restricts the promoter activity of matrix metalloproteinases‐9 and the expression of protein via aiming Sp1. The topical application of these miRNAs in diabetic animal models ascertained their valuable contribution to diabetic wound repairing. This therapeutic approach the expression of matrix metalloproteinases‐9 mediated by Sp1 and enhanced the migration of keratinocytes and collagen content.^120^

During the remodeling phase, the blood vessels toward the wound site reduce, and the cells' activities begin to slow down in preparing for termination. In particular, miRNA‐29a regulates the fibroblast by monitoring their contractility via targeting tissue inhibitors of metalloproteinase‐1.^121^ Besides this, miRNA‐29a has also been verified to have a direct impact on collagen expression. Additionally, miRNA‐29b, ‐29c, and ‐192 are extremely stimulated in this phase.^122^, ^123^ Out of all the stages of wound healing, this stage demands additional research into finding the different miRNAs engaged with its regulation.

## 4 miRNA AND THE ORAL MUCOSA WOUND HEALING PROCESS

Skin and oral mucosa display various structural and functional similarities, with both consisting of a keratinized epithelium on an underlying type I collagen‐rich connective tissue, which plays a vital role as a barrier to microorganisms and external stimulus. Furthermore, the skin and oral mucosa share many structural and functional wound healing similarities. The skin and oral mucosal wound healing process include similar consecutive stages of hemostasis and inflammation, proliferation, and remodeling. Briefly, hemostasis is obtained via fibrin‐rich granulation tissue formation, which matures in the inflammatory phase. Epithelial cells proceed to the wound base at the termination of the inflammatory phase, initiating wound transition into the proliferative phase. Finally, fibroblast extracellular matrix secretion and granulation tissue remodeling approximate the tissues' structure and function.^124^, ^125^

**FIGURE 3:**
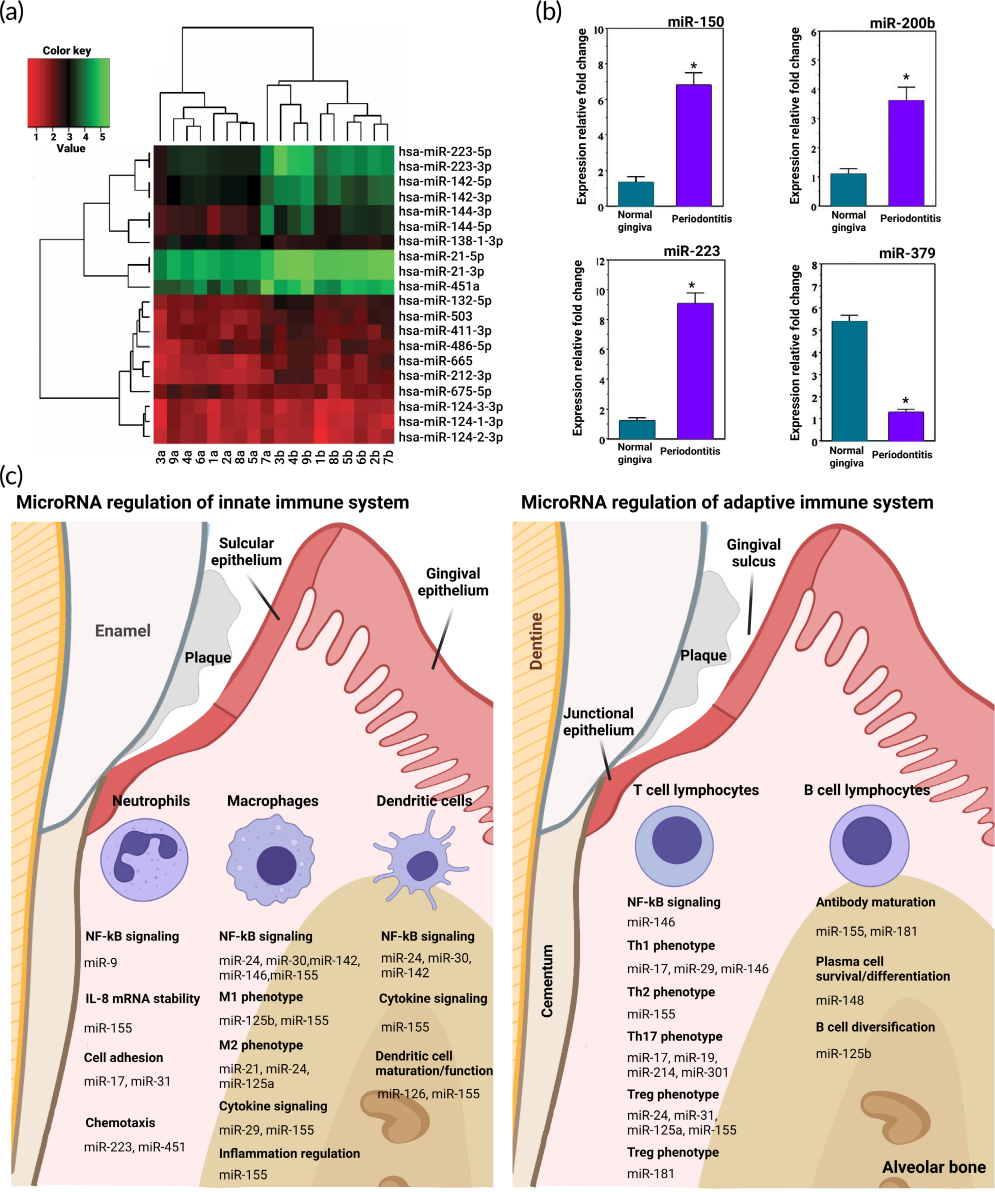
miRNA plays a role in the periodontal tissue regeneration process. (a) The top 20 most variated miRNA expression profiles in day 5 of human gingiva wound healing. 1a–9a displays healthy gingiva, and 1b–9b displays the healing gingiva of the identical person. The colors of this heatmap exhibit the upregulation and downregulation with green and red, respectively. Reproduced from Reference 127 with permission from Wiley. (b) The expression of miR‐150, miR‐200b, miR‐223, miR‐144, miR‐379, and miR‐222 in three samples of inflamed and noninflamed gingiva using real‐time PCR (*p < 0.01). Reproduced from Reference 128 under open access license. (c) Schematic on the regulative role of miRNA (i.e., promote or inhibit) on the innate immune cells (i.e., macrophages, neutrophils, and dendritic cells), besides adaptive immune cells, including T and B lymphocytes.^129^ IL, interleukin; miR, miRNA

The gingiva is a component of the oral mucosa and comprises dense connective tissue and oral epithelium. Gingivitis, the primary prevalent form of gingival disease, is attributed to the inflammatory condition originating from dental biofilm accumulation and diagnosed by gingival edema, redness without sign of periodontal attachment loss.^126^ Mechanisms underlying tissue response to the inflammation are still not completely recognized. Recently, miRNAs expression profile and their potential role in gingival wound healing were investigated through a human experimental study. The results demonstrated that relatively few miRNAs are diversely expressed throughout the progression from the inflammatory to the proliferative stage in the healing gingiva. The results also showed the possible role of hsa‐miR‐124‐3p as a critical regulator of gingival healing in contact with angiogenesis. Figure 3a represents the miRNA expression profile through the initial wound healing in human gingiva, indicating the variation in miRNA expression profile in wound healing gingiva compared with normal gingiva.^127^ In addition, specific miRNAs are suggested to have the potential to ameliorate the oral wound healing process. The positive effect of miRNA‐21 in palatal wound healing has been evaluated on a mice model. The results showed that miRNA‐21 enhances oral wound healing through increasing extracellular matrix production with the inhibition of autophagy.^130^

Periodontitis is a complex inflammatory disease of the teeth supporting tissues, causing progressive destruction of periodontal ligament and alveolar bone. Generally, gingivitis precedes periodontitis, and in gingivitis, the inflammation is restricted to the gingiva; however, in periodontitis, the inflammation extends to periodontal tissues.^126^ Bacterial exposure has long been considered a prerequisite for periodontal inflammation, in which microbial biofilm on the enamel surface and in the gingival sulcus triggers the periodontal immune response. Bacterial exposure is now understood to be necessary for the onset of periodontal disease, but the host response ascertains the disease phenotype. Genetic polymorphisms and epigenetic varieties, such as miRNAs, can influence innate and adaptive immune systems response, resulting in differences in individual responses, modulation of clinical manifestations of inflammation, and distinctive treatment responses.^131^, ^132^, ^133^

Multiple human experimental studies have evaluated miRNA expression patterns in healthy and diseased gingival tissues. These miRNA profiling studies indicated that periodontal disease cause alteration in miRNA expression profile in diseased tissues.^134^, ^135^, ^136^ The miRNA expression profile in the healthy gingival tissues and periodontitis was compared, and the results indicated upregulation in miR‐223, miR‐150, miR‐200b, and miR‐144 besides downregulation in miR‐222 and miR‐379 (Figure 3b).^128^ The serum and gingival cervicular fluid miR‐223, miR‐203, and miR‐200b relative quantities was assessed in systemically healthy patients with chronic periodontitis and type 2 diabetic patients with chronic periodontitis. The results indicated a significant increase in miR‐223 and miR‐200b expression profile during chronic periodontitis with and without type 2 diabetes highlighting their role in chronic periodontitis besides their potential as chronic periodontitis serum biomarker. Furthermore, there was a significant decrease in miR‐203 expression profile with negative correlation with inflammatory markers that accentuate its potential role in chronic periodontitis protection and healing.^137^

The oral epithelium and immune cell populations found in periodontium serve as the first line of defense against oral bacteria and contribute to the primary host response toward infection.^138^ The innate immune response is not directed against specific pathogens but instead provides a nonspecific front line of defense during the first hours after infection. In addition to the oral epithelial cells that act as a barrier against external stimuli such as toxins and pathogens in the oral cavity, the principal cells implicated in the immune response are neutrophils, macrophages, and dendritic cells.^139^, ^140^ Adaptive immunity is characterized by a tightly regulated interaction between antigen‐presenting cells and T and B lymphocytes, allowing pathogen‐specific elimination or growth inhibition.^141^ miRNA levels alteration impacts the innate and adaptive immune responses of the host toward bacterial infection in periodontal tissues (Figure 3c).^128^

**FIGURE 4:**
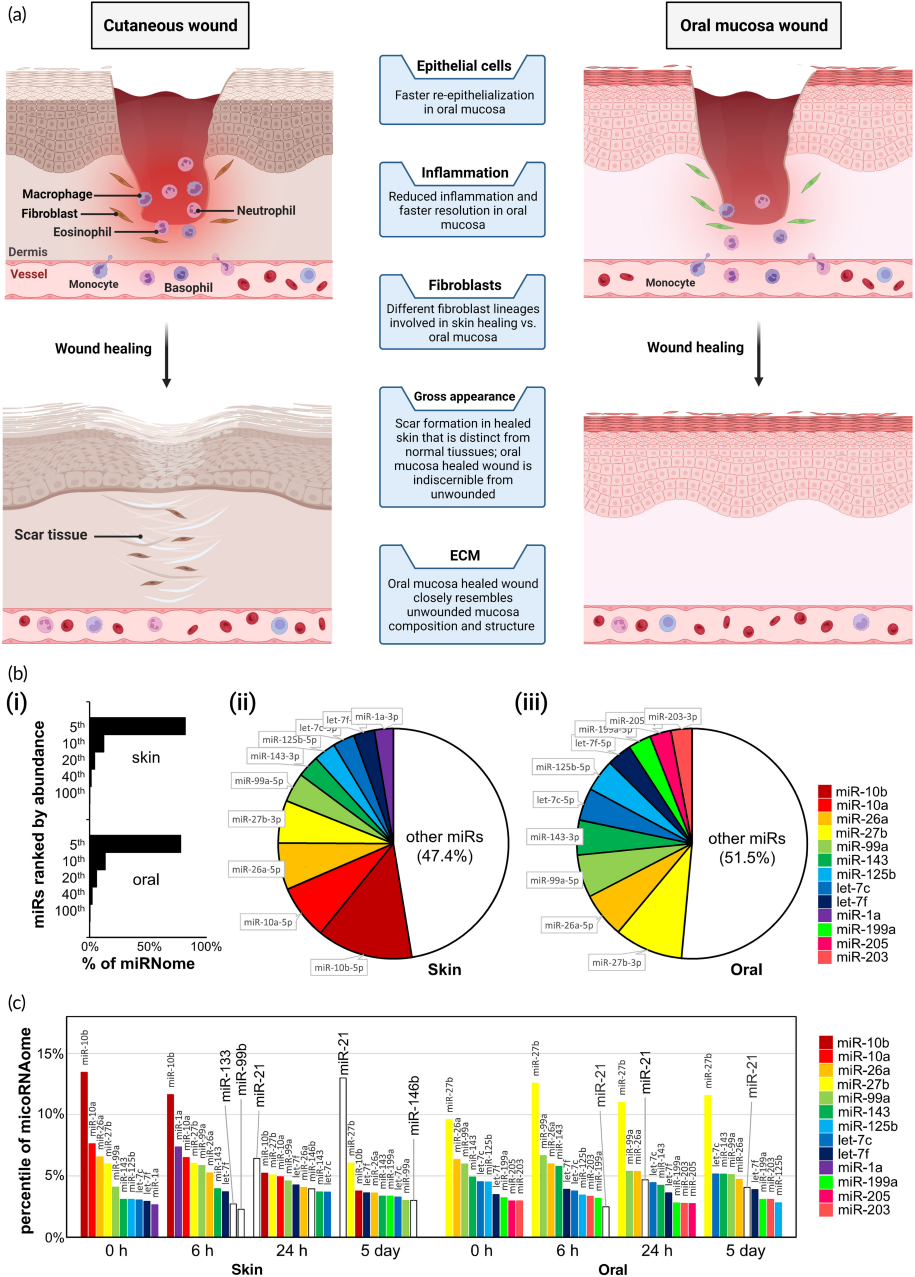
Comparison between the skin and oral mucosa in normal condition and during wound healing. (a) Schematic on the major differences of skin and oral mucosa wound healing process. (b) miRNA expression profile of skin and oral mucosa in the normal condition; (i) applying miR‐Seq analysis, the top 5% most plentiful miRNA species calculated 81.64% for skin epithelium and 77.8% for oral mucosa epithelium. (ii) The top 10 mostly expressed miRNA in the skin (iii) and oral mucosa (palate). (c) miRNA expression profile variation within the skin and oral mucosa wound healing process. Variation in the expression level of 10 primarily expressed miRNAs of skin and oral mucosa through the wound healing process, displayed as the percent of the miRNAome. miR, miRNA. Parts (b) and (c) are reproduced from Reference 142 under the terms of CC‐BY license open access

Despite several similarities, oral mucosa wounds possess preferable healing characteristics compared to skin wounds since they heal faster with minimal scar tissue formation (Figure 4a).^143^ Oral mucosa wounds undergo a healing process in an environment that withstands continuous physical trauma and high level of bacteria.^144^ Accordingly, environmental differences (i.e., temperature, saliva, and microflora) were offered as the origin of the variations in wound healing profile. However, cutaneous grafts transposed into the oral cavity preserve their morphology and healing characteristics. These findings unveiled that premiere wound healing of oral mucosa is related to the intrinsic properties of oral mucosa instead of environmental factors. Regarding the intrinsic properties, recent studies highlight the role of various mechanisms at the cellular and molecular level, including distinctive inflammatory responses, different modulation of stem cells, proliferation and differentiation mechanisms, and more effective epithelial remodeling. Nevertheless, many of the molecular mechanisms underlying wound healing in both skin and oral mucosa are not entirely characterized, which hardens their comparison from this point of view.^142^, ^145^, ^146^

So far, the intricacy of the wound healing process at the genomic extent is distinctly indicated. A comparison between miRNA expression patterns of skin and oral mucosa at normal and wound healing states was conducted. The results displayed the baseline diversity of the site‐specific miRNA profile in healthy skin and oral mucosal epithelium. Furthermore, the miRNAs expression pattern was also different with oral mucosa over time, consistent with the variation in wound healing, and implies that the oral mucosa holds an intrinsic and modified genetic reaction that enhances wound healing (Figure 4b,c). Additionally, the application of miRNA for accelerating skin wound healing also has shown promising outcomes. The manipulation of miRNAs accelerated skin wound healing, offering the practicability of miRNA in remedial attitudes to enhance wound closure and prevent chronic wounds.^141^

## 5 TARGETED miRNA DELIVERY FOR CHRONIC WOUNDS

Since miRNAs are implicated in the regulation of numerous conserved cell‐signaling pathways, altered expression of miRNAs could be involved in different pathological responses. In this regard, the fine regulation of miRNAs expression profiling is crucial in diverse biological processes.^147^ To date, many studies have manipulated the miRNA's expression profiles for therapeutic purposes. Due to the unstable nature of miRNAs molecules and their susceptibility to degradation by nucleases, the development and optimization of efficient delivery methods are a major challenge in miRNA‐based therapeutic strategies. In the following sections, we highlight the various delivery vectors with a focus on the viral and nonviral miRNA delivery systems for wound healing.

### 5.1 Viral vectors

**FIGURE 5:**
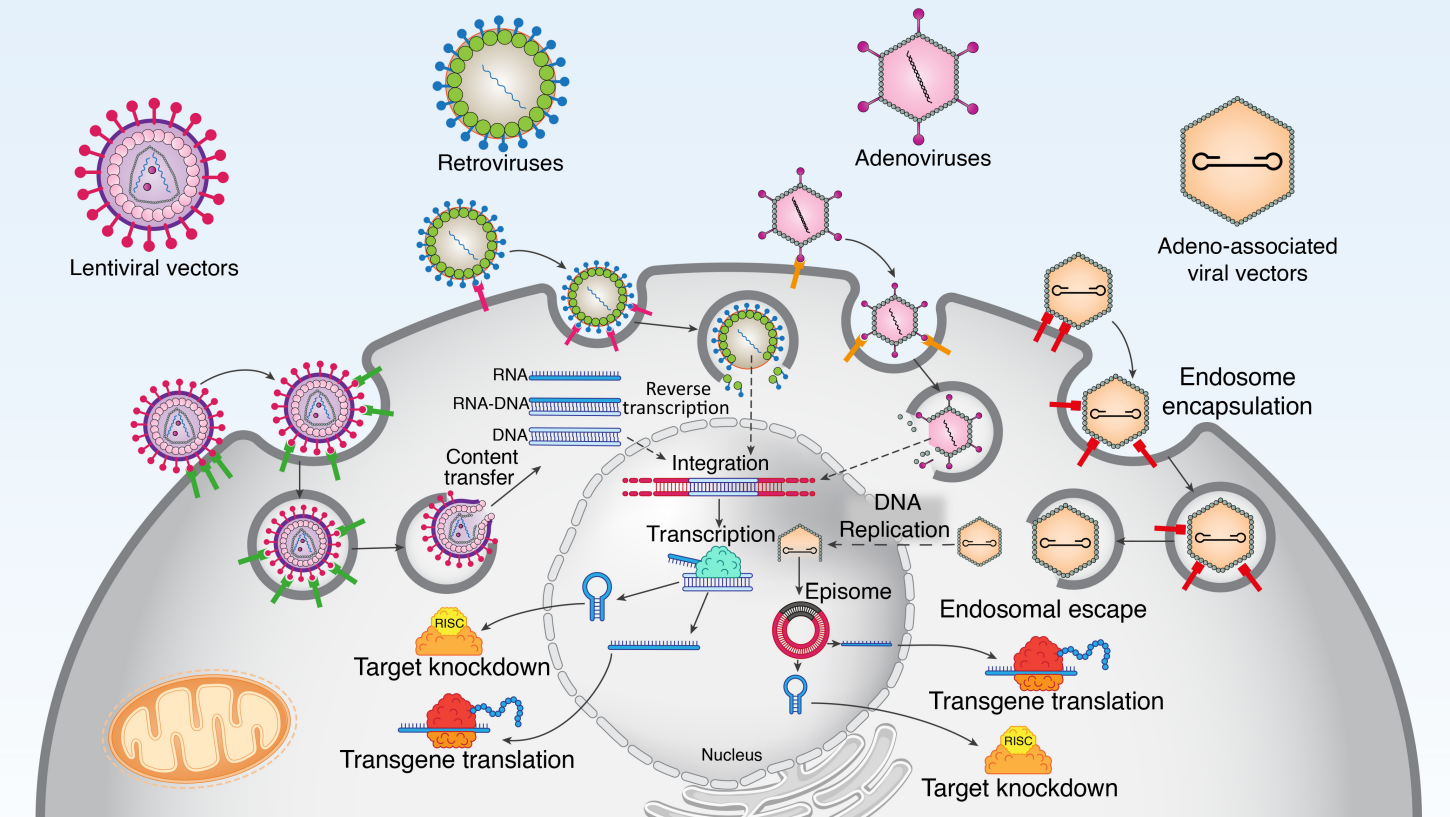
Schematic illustration on viral‐based transgene delivery mechanism for gene therapy. In viral‐based gene delivery systems, genetically engineered viruses are used to infect target cells for gene transfer. Nuclear entry is followed by efficient transgene expression, introducing the viral vectors as powerful vehicles for gene transfer. Abbreviations; RISC: RNA‐induced silencing complex. 147

Targeted gene transfer and efficient gene expression can be achieved utilizing viral vectors. They are advantageous due to their increased transduction or transfection efficacy over a broad array of human cells. This ability made them attractive vehicles for the delivery of therapeutic genes (Figure 5).^148^

Numerous viral vectors with different genomic structures and host ranges are designed and developed for laboratory and clinical usage. Since different types of viral vectors possess unique properties and have specific advantages and limitations, certain vectors are often preferred for definite objectives.^149^ Adenoviruses and adeno‐associated viruses, lentiviruses, and retroviruses are the most frequently used vectors for targeting skin tissues. These viral vectors are engineered in some particular genomic region so that they are unable to replicate in the target cell, and their safety is increased.^150^

Retroviral vectors are frequently employed to deliver miRNAs expression cassette into somatic and germline cells. After virus entry, double‐stranded DNA is formed and then integrated into the host genome during the mitotic phase of the cell cycle, which leads to the persistent expression of the exogenous sequence and stable expression of miRNA.^151^ To improve the production efficiency of pluripotent stem cells, retroviral infection was used for miR‐138 induction more than 1000‐fold in mouse embryonic fibroblasts.^147^

Adenoviruses are double‐stranded DNA viruses, which are unable to integrate the exogenous gene fragment into host genomic DNA and remain episomal. This feature makes adenovirus vectors an ideal gene delivery system for treatment strategies that need high but temporary gene expression. In addition, their episomal survival in target cells enhances the safety profile of these vectors for gene transfer in clinical studies.^152^ Adeno‐associated virus, in contrast to adenoviruses, is more commonly utilized in transferring miRNAs due to the low loading capacity.^147^ Gene delivery mediated by viral vectors is assessed on the cutaneous wound healing in animal models. Mounting evidence highlight the role of VEGF in wound repair. To investigate the therapeutic effect of VEGF expression during wound repair, a recombinant adenovirus vector carrying the coding sequence of VEGF was applied on excisional wounds of diabetic mice. The results show the potency of adenovirus vector in VEGF gene delivery as well as the potential benefits of gene therapy in wound healing.^153^

**FIGURE 6:**
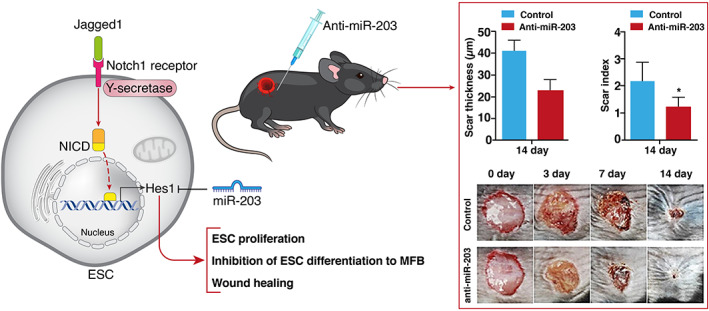
Notch/ Jagged1 signaling is a major regulator of epidermal stem cell proliferation differentiation through Hes1 expression. Hes1 downregulation via miR‐203 could be a major molecular mechanism in wound healing and scar formation. Anti‐miR‐203 treatment promoted wound closure and decreased scar formation rate in vivo. Direct injection of the anti‐miR‐203 into the tissue around the wound resulted in a significant reduction in scar thickness and index compared to the control group at day 14. *p < 0.05. ESC, epidermal stem cell; Hes 1, hairy/enhancer of split‐1 gene; MFB, myofibroblasts; NICD, notch intracellular domain. Right panel was reproduced from Reference 117 under open access license

Lentiviruses, as a subgroup of retroviruses, can infect both dividing and nondividing cells and result in the high transfection rate and long‐term expression of introduced miRNAs. Also, lentiviral‐based miRNA target gene expression was used to determine the role of miRNAs regulatory effect in wound healing. In a recent study, a lentiviral construct was used to express the Hairy/Enhancer of split‐1 gene, which is targeted by miR‐203, in epidermal stem cells. The results from this study indicated that anti‐miR‐203‐based treatment improved wound healing and reduced scar formation in vivo (Figure 6).^117^ Moreover, lentiviral vectors have also been implicated in the upregulation of specific miRNAs expression, which provides a detailed view of the regulatory mechanisms of miRNAs in the wound healing process. Previous studies demonstrated markedly impaired angiogenesis signaling in diabetic wound lesions. Computational analysis candidate the globin transcription factor binding protein 2 and VEGF receptor 2 as potential targets of miR‐200b in the angiogenesis pathway. In vivo dermal injection of miR‐200b overexpressing lentivirus show the efficacy of targeting miRNAs inducing pathways in wound healing.^154^

Although viral vectored delivery of miRNA has proven to be efficient, some limitations restrict their usages, such as insufficient loading capacity, induction of immune system, off targeting, toxicity, and risk of insertional mutagenesis. Subsequently, several nonviral vector delivery systems have been designed to overcome these obstacles. Their minimal toxicity and ideal biocompatibility bring the nonviral vectors forward as a valuable gene delivery method. However, due to the lower transfection efficiency of the non‐viral‐based gene transfer strategies, the number of viral vectors as an efficient delivery platform for genetic manipulation of dividing and nondividing cells is increasing.^155^, ^156^

### 5.2 Nonviral vectored delivery systems

Poor cell membrane permeation (due to higher molecular weight and negative charge), short half‐life in blood circulation, and quick removal of the biomolecules from the plasma are the major hurdles that obstruct the fortunate transport of the miRNAs^157^ Consequently, developing an effective and secure carrier system for delivering miRNA to the target tissue is of paramount importance.^158^ As an alternate, the nonviral delivery vehicles incrementally happened to be the major purpose of the study.^159^, ^160^ Unlike viral vectors, nonviral systems are accentuated by low immunogenicity and toxicity profile, high cell uptake, aqueous solubility, and elicit resistivity toward endonucleases and phagocytosis.^161^, ^162^, ^163^, ^164^ Gene transfer via a nonviral vectored system is accomplished by establishing the desired plasmid RNA‐encoded gene, RNA interference, and miRNA. Different nonviral vectors have been developed so far and some of them include cationic polymers, lipid‐based carriers, micro‐seeding, naked plasmids, dendrimers, electroporation, and particle bombardment.^165^, ^166^, ^167^ However, the role of not all the nonviral vectored systems developed to date is clear in the case of chronic wound healing. At present, the lipid‐derived natural and synthetic polymers, for instance, chitosan and phosphatidylcholine have gained popularity and are used frequently as a nanocarrier for miRNA transport. These vectors once reaching the target site are biodegraded to give products that could be easily absorbed by the distinct biochemical pathways in the human body.^168^ Nevertheless, these genes transfer approaches also demonstrate potential healing actions in animal studies.

Generally, based on the nature of the approach, nonviral systems can be mainly divided into three classes, namely complexation, conjugation, and encapsulation.^158^ The term complexation applies to abridging the negatively charged miRNA by the positively charged carrier systems resulting in the formation of polyplexes through electrostatic linkages. Among several liposomes, which consist of a phospholipid bilayer membrane, are the most popular miRNA complexations in the market. In the case of conjugation, there is covalent binding of miRNA with the vectors through linkers resulting in a delivery system with superior stability and the ability to protect the miRNA in the blood circulation. The third category is encapsulation, which refers to loading the miRNA molecule in a biodegradable NPs that further carries it to the desired site. Poly(lactic‐co‐glycolic acid), silica NPs, and inorganic NPs containing gold are some of the widely employed NPs these days. Table 2 represent different nanoplatforms for delivery of miRNA.

**TABLE 2:** Different delivery platforms for miRNA and the possible vectors

miRNA	Type of miRNA	Delivery device	Ref
miRNA‐155	Anti‐miRNA	Lipid NPs	169
miRNA‐210	Anti‐miRNA	Lipid NPs	170
miRNA‐21	Anti‐miRNA	Dendrimer	171
miRNA‐29b	miRNA mimic	Cationic lipoplex	172
miRNA‐34a	miRNA mimic	Silica NPs	173
miRNA‐155	Anti‐miRNA	Polylactic glycolic acid NPs	174
miRNA‐21	Antisense	Polyethleneimine‐polylactic glycolic acid NPs	175
miRNA‐34a	miRNA mimic	Liposome	176
miRNA‐221	Anti‐miRNA	Magnetic NPs	177
miRNA‐29	Anti‐miRNA	Gold NPs	178
miRNA‐542‐3p	miRNA mimic	Polyethleneimine‐polylactic glycolic acid NPs	179
miRNA‐21	Anti‐miRNA	Graphene oxide nanocomplex	180

#### 5.2.1 Inorganic nanocompunds

In the past couple of decades, NPs have grown to be an influential biological vehicle in the domain of regenerative medicine, and their diminished size ranging in <1 μm has made them different from the other delivery vectors.^181^, ^182^ The unique features of NPs, including electrical conductivity, solubility, biological distribution, antimicrobial actions, immunogenicity, contractility, swelling, and higher surface–volume ratio establish these minute systems as an attractive and fascinating drug transport vehicle for transdermal as well as topical practice. Moreover, these strategies hold the caliber of circumventing the disappointments of conventional treatment options in regenerative medicine.^183^, ^184^, ^185^ NPs bring about an improvement in the miRNA distribution in the body and its localization in specific tissues. However, the degree of enhancement, in general, continues to be inadequate. As a result, innumerable studies are focusing on developing NPs with surface modification using ligands to obtain better specificity toward the targeted receptors on the cells, hence making the receptor‐mediated endocytosis an easier task for uptake of NP together with decreasing the essential dose and the associated adverse effects of the regimen.^186^, ^187^ Additionally, the NPs colloidal stability in a complex physiological environment is required for the miRNA delivery toward the target cells.^188^

Following administration, NPs must preferably distribute if they arrive at the target site. They must be designed so that they undergo endosomal escape to ensure the suitable miRNA interaction with its target (for instance, by utilizing the Proton Sponge Effect).^189^ However, the circulation time is dependent on the interaction of NP with the biological environment, which might further result in a faster clearance.

The biocompatibility and biodegradability of nanomaterials make them an ideal nanocarrier for drug delivery. Consequently, nanomaterials are highly safe in vivo. Their biocompatibility and biodegradability are high since they are the principal components of biological tissues and participate in the body's natural metabolism through dissolution into nontoxic ions.^190^ Usually, NPs are expelled out of the body in two main ways that are through the urinary system and through the hepatobiliary system and feces. Heavy metals and particles with a diameter larger than 6 nm can be quickly absorbed by the liver and spleen. Additionally, NPs with sizes less than five nanometers are rapidly metabolized in the urine by the renal system because their filtration sizes are smaller than those required for renal excretion.^191^

In recent times, NPs derived from inorganic materials such as gold NPs, graphene oxide, mesoporous silicon, and ferric oxide NPs are exploited as a promising vehicle for miRNA delivery in chronic wound healing. Gold NPs offer an easier functionalization with numerous biomolecules, including amino groups and thiols. Experimental data have frequently proven these gold NPs to be nonimmunogenic and have a clear cytotoxic profile.^192^ An amino‐functionalized gold NPs was designed to deliver miRNAs by first complexing the miRNA with gold NPs followed by coating it with polyethylene glycol. The outcomes of the study concluded that the system could undergo effective and easy cellular uptake via endocytosis along with the timely release of the miRNA complex at the site of interest and was nontoxic to the cells.^193^ In another study, covalent conjugation of gold NPs was fabricated with antagomir‐miRNA‐155 and thiol modification. The intravenous injection of the miRNA‐155 loaded gold NPs in an ovariectomized diabetic murine model fostered the polarization of M2 macrophages and at the same time lowered the inflammatory mediators, subsequently resulting in the recovery of cardiac functionalities.^194^

A magnetic nonviral delivery vehicle was developed for effective miRNA transport in human mesenchymal stem cells isolated freshly. Multiple miRNAs and polyethyleneimine complexes loaded in magnetic NPs were examined to allow magnetic targeting of transfected CD105+ human mesenchymal stem cells, gaining higher uptake rates with negligible toxicity.^195^ Likewise, a nonviral and nonlipid scaffold approach was presented for the very first time to deliver both mimics as well as anti‐miR to the human mesenchymal stem cells. In the experiment, nanohydroxyapatite particles were developed that successfully delivered miRNAs to the human mesenchymal stem cells in both 2D and 3D culture media, thus establishing this strategy as a potential therapeutic choice for a broad spectrum of tissue repairing applications.^196^

**FIGURE 7:**
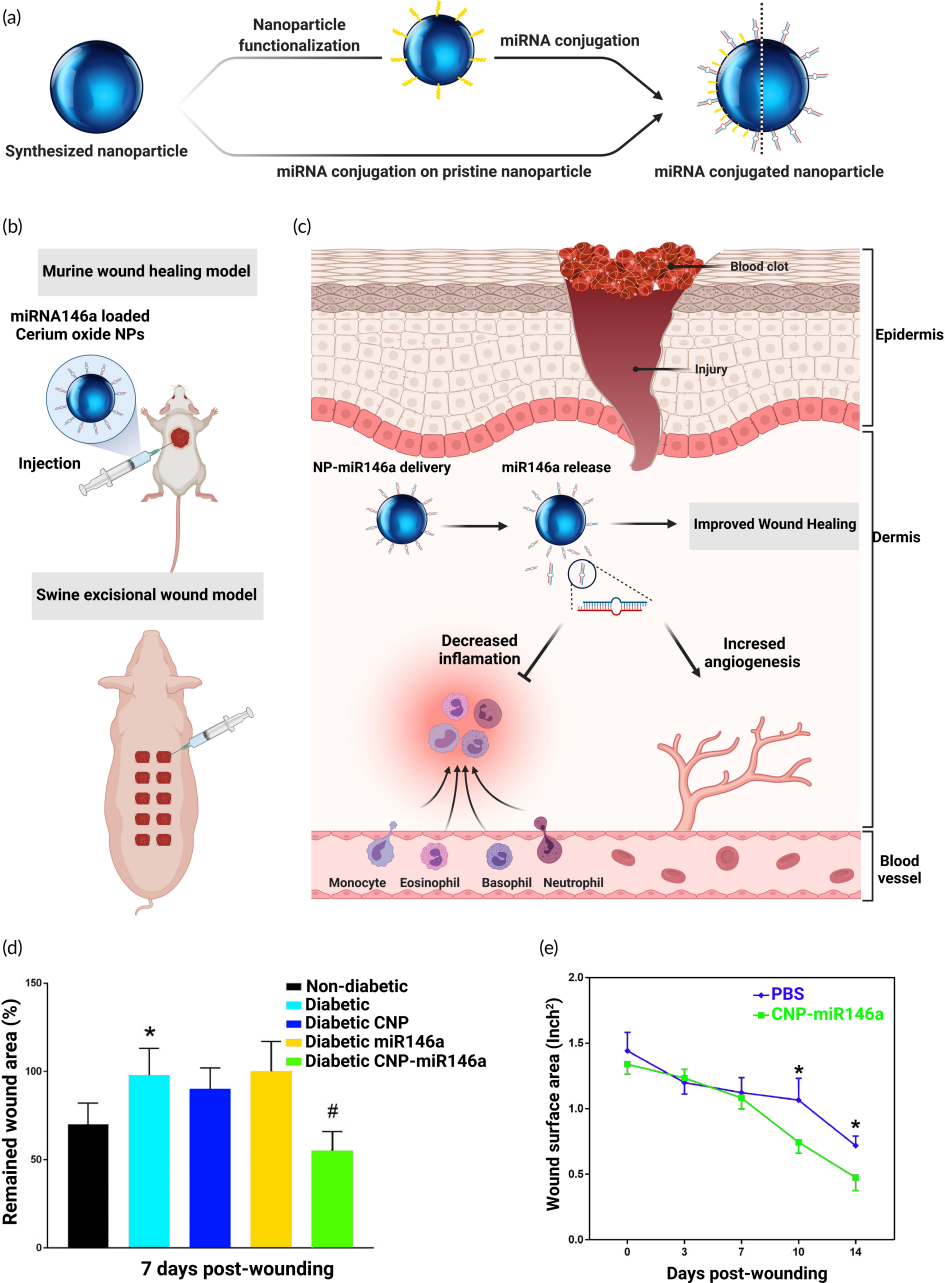
miRNA conjugated metallic NPs enhance wound healing. (a) Schematic on the preparation of miRNA conjugated NPs. (b) Delivery of miRNA‐146a loaded cerium oxide NPs using an injection in a swine excisional wound model showed decreased inflammation and increased angiogenesis. (c) Graphical representation showing application of NP mediated delivery of miR‐146a, its release and improvement in wound healing by means of decreased inflammation and increased angiogenesis. (d) Graphical representation showing nondiabetic wounds treated with PBS, diabetic wounds treated with PBS, and diabetic wounds treated with 100 ng CNP, 106 PFU LentimiR‐146a, or 100 ng of CNP‐miR‐146a at day 7 post‐wounding. (e) Representation of wound closure concerning the time of PBS and CNP‐miR‐146a treated wounds. The comparison was performed between PBS and CNP‐miR‐146a treated wounds. CNP, cerium oxide NPs; PBS, phosphate buffer saline. Parts (d) and (e) are reprinted from Reference 198 with permission from Elsevier

An experimental study carried out by Krebs research group is of paramount importance in this regard. The Krebs and Liechty laboratories have notably concentrated on the miRNA‐146a mimic since it has been observed that miRNA‐146a regulates the inflammatory milieu in diabetic wounds. It is believed to work by aiming a target on the key adapter molecules present in the NF‐κB inflammatory signal transduction pathway, thereby causing a reduction in the expression of the pro‐inflammatory cytokines, including the interleukins.^197^, ^198^ Since there is a downregulation of miRNA‐146a in diabetic wounds, Liechty's research group injected a combination of the miRNA with cerium oxide NPs as a reactive oxygen species scavenger that may aid in the reduction of total inflammatory condition and thus bring about an improvement in the wound healing process. During the wounding phase, the mice were provided with a single treatment, and the kinetics of release was not noted in the study. A considerable reduction in the wounded area was witnessed at the interval of 7 days of miRNA‐cerium oxide NPs administration. This outcome highlighted the significance of an appropriate carrier system for miRNA delivery (Figure 7).^198^, ^199^

Furthermore, the miRNA‐146a‐cerium oxide NPs were laden in a zwitterionic hydrogel system to present a persistent release for almost a month, with approximately 50% of the NPs released in the first couple of days. In in vivo studies, the release of miRNA‐146a‐cerium oxide NPs from the hydrogel elicited a complete wound closure by 2 weeks in contrast to around 20 days in the case of mice solely treated with hydrogel.^200^ Apart from these inorganic material‐based NPs, poly(lactic‐co‐glycolic acid) NPs are also being exploited as miRNA delivery vehicles on a larger basis. Poly(lactic‐co‐glycolic acid) is a polymer synthesized by copolymerization of glycolic acid and lactic acid and owns biodegradability besides biocompatibility.^158^ The intracellular miRNA delivery can further be improved using poly(lactic‐co‐glycolic acid) NPs after ornamenting them with peptides with cell‐penetrating properties.

Based on those mentioned earlier and several other experimental outcomes, it can be proposed that compared to free molecules, biomolecules delivered using NPs demonstrate better effects in chronic wound healing.^201^, ^202^, ^203^ There are, however, some limitations to NP‐based delivery systems, such as the development of complex and rapid clearance. Specifically, the gold NPs possess detrimental properties such as poor encapsulating performance, delayed endosomal escape, and stability issues in storage. Moreover, knowledge regarding their ability to safeguard their payload from in vivo degradation draws a line in their applications. Nonetheless, before their use in clinical settings, greater perceptions are required regarding the appropriate targeted delivery, including advanced research to improve our knowledge about off‐target consequences.

#### 5.2.2 Lipid‐based NPs

Lipoidal vesicles, which are pockets consisting of phospholipid bilayer membrane enclosing an aqueous chamber,^204^ are the finest candidate vehicles for the complexation of miRNA. In the delivery of miRNA, lipid vesicles either undergo chemical modification with target‐specific groups or are PEGylated to prevent recognition by the immune system and reticuloendothelial system uptake.^158^, ^205^ Cationic lipids, which are amphiphiles consisting of a hydrophilic head and lipophilic tail,^206^ are recently elected from commercial products like Lipofectamine®. Several research investigations have suggested the in vivo utilization of cationic liposomes as vehicles for miRNA delivery. These systems display benefits in many areas, especially in the effortless formation and significant interaction with the negatively charged biological membranes. However, poor delivery performance and higher toxic effects are the major barriers that obstruct the clinical use of lipid‐based delivery approaches. Despite this, a cationic lipoplex‐based system for delivering miRNA was developed which significantly enhanced the transfection efficacy than the standard delivery agents available in the market (siPORT, NeoFX transfection agent, Ambion), in vivo as well as in vitro.^169^

**FIGURE 8:**
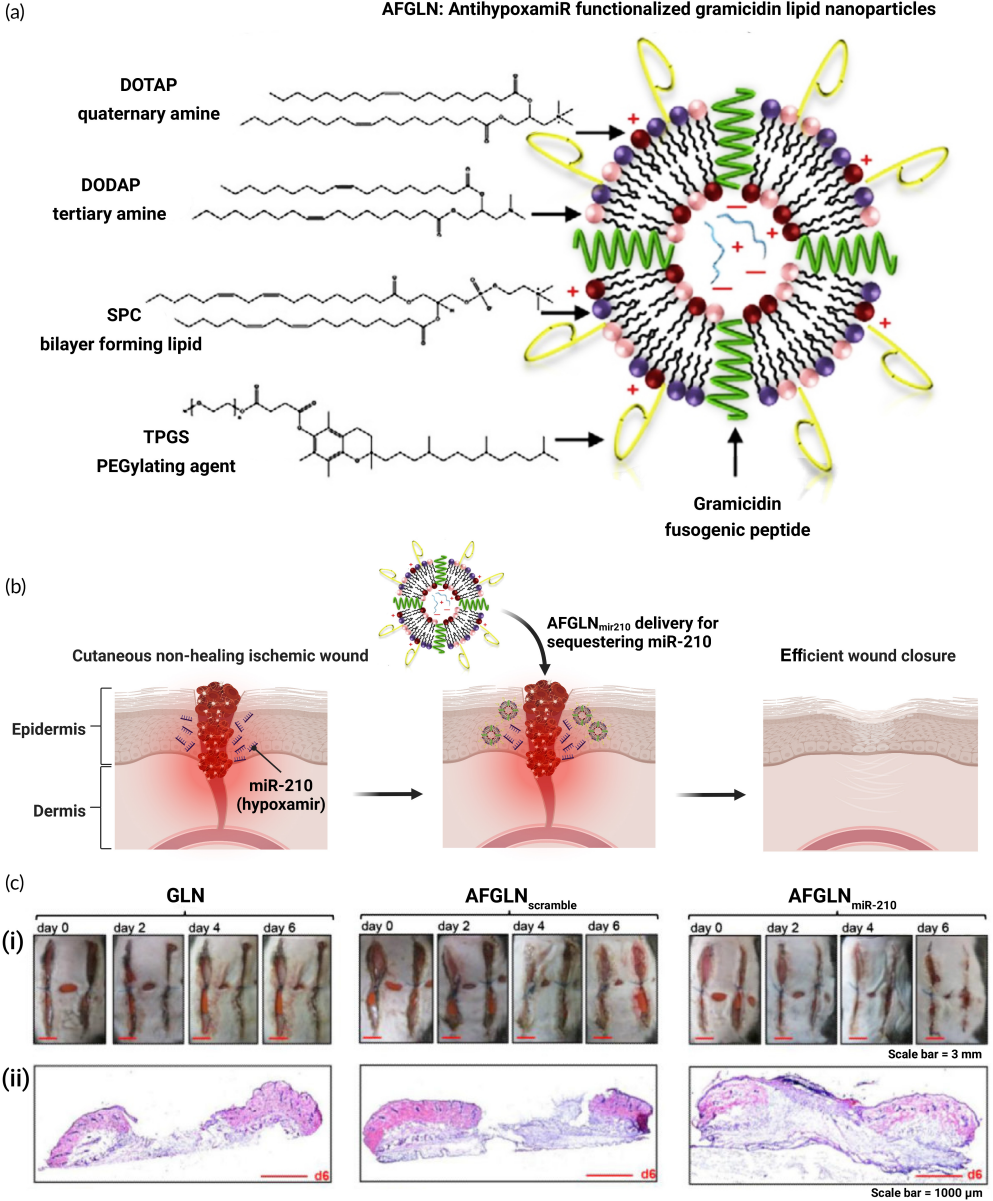
Schematic representation of antihypoxamiR lipid NPs for cutaneous wound healing. (a) Schematic representation of the functionalization of AFGLN. (b) Delivery of AntimiR‐210 using AFGLN improved ischemic wound closure. (c) Digital photographs showing (i) murine ischemic wound at days 0, 2, 4, and 6 days after delivery of nascent GLN, AFGLN scramble, and AFGLNmiR‐210. (ii) Hematoxylin and eosin (H&E) stained sections from the ischemic wounds at day 6 post‐wounding. AFGLN, AntihypoxamiR functionalized gramicidin lipid NPs; GLN, gramicidin. Reprinted from Reference 170 with permission from Elsevier

Lipid NPs have also turned out to effectively deliver miRNAs to the targeted location in the body. An antihypoxamiR laden lipid NPs was fabricated focusing on promoting subcutaneous wound healing. Lipid bilayer formation was achieved using soy phosphatidylcholine, cationic lipids having tertiary DODAP and quaternary DOTAP amine lead groups, and Gramicidin A to enhance the endosomal escape together with facilitating the formation of ionic channels in the bilayer (Figure 8a). Intradermal injection of these NPs in the bipedicle flap wounds generated in atherosclerosis and diabetes‐prone mice showed significant downregulation in the expression of miRNA‐210 in the ischemic wound edge tissue (Figure 8b).^170^ It was found that wounds treated with empty GLN 113 were found to compromise wound re‐epithelization severely alone or AFGLN packed with scrambled 114 oligos (AFGLN scramble) (Figure 8c). Additionally, the utilization of Lacto‐bionic acid for partial reduction of positive charge on the surface of liposomes ultimately resulted in reduced toxicity of lipid vehicles for delivery of miRNA.^169^ These outcomes state that liposomes continue to be a potential miRNA transport system if safety concerns are resolved. To defeat these limitations, novel lipids are being synthesized and newer techniques are adapted for building lipoidal nanocomplexes.

#### 5.2.3 Organic‐based nanomaterials

Natural and synthetic polymers are perceived as another potential material for miRNA delivery on account of greater adaptability and flexibility. Polymer's facile size and charge adjustability are authorized to optimize the loading potential for nucleic acids.^158^ Conventionally, Polyethylenimine is the most popular synthetic cationic polymer for the delivery of nucleic acids. They possess abundant amine moieties and carry a positive charge. Therefore, they can bind to the smaller RNAs to create nanoscale complexes that obstruct the degradation of RNA and thus enhance the uptake by the cells and promote its release intracellularly.^207^ Additionally, the myriad of tertiary amines in polyethylenimine greatly expedites miRNA‐loaded polyplexes' endosomal escape through the proton sponge effect.^208^ Recently, linear or branched polyethylenimine having distinct molecular weight falling in the range 100 Da to 1000 kDa approximately can be procured.^209^ Preliminary investigations have demonstrated that 25 kDa polyethylenimine with branching was more potent in transporting mmu‐miRNA‐494‐3p in embryonic fibroblast cells in mice than Lipofectamine 2000.^210^

Chitosan is another linear biopolymer that has been frequently utilized for gene transport. It comprises an amino moiety and two hydroxyl groups within the glucoside residue. Furthermore, the preparation method can affect its structure.^211^, ^212^, ^213^ Chitosan, as a cationic polymer plays an important role in providing some fascinating characteristics regarding oppositely charged units, particularly, nucleic acids and surfaces of mucosa (through sugar moieties like sialic acid).^214^, ^215^ Together with lower immunogenicity, biodegradability, and biocompatibility, these exceptional characters present chitosan as a better agent for gene delivery in bedside use.^13^, ^216^ Several derivatives of chitosan can be employed for the targeted biomolecular delivery. Chitosan with cationic polyelectrolyte character offers a stronger electrostatic interaction with the negatively charged nucleic acids and thus safeguards it from degradation by nuclease.^217^, ^218^ Nevertheless, due to its poor transfection efficacy, the in vivo applications of this polymer are restricted. As a result, scientists used different strategies to enhance the biomolecular transfection efficiency of chitosan.

#### 5.2.4 Dendrimers

As an alternative to the previously known polymers, cyclodextrin has grabbed much of the attention in the delivery of biomolecules.^219^ As a form of naturally found cyclic oligosaccharide, cyclodextrin is expressed as nonimmunogenic and nontoxic to cells.^220^ These properties have made polyethyleneimine quite popular in reducing the toxicity of polymers such as polyethylenimine toward cells in nucleic acid delivery through covalent modifications in miRNA conjugation. Polyethylenimine implant was synthesized to bestow favorable characteristics related to polyethyleneimine (nontoxic effects, higher aqueous solubility, ability to form inclusion complexes) to polyethylenimine. Following several investigational studies, it is understood that the finest cyclodextrin‐branched polyethyleneimine for delivery of plasmid was fixed to hold 8% PEI‐amine implant, whereas the finest cyclodextrin‐linear polyethylenimine was synthesized with 12% implantation. Upon testing of these finest cyclodextrin‐linear polyethylenimine polymers for their potential to deliver plasmids to the PC3 cells, it was observed that cyclodextrin‐linear polyethyleneimine polymers were more efficient in delivering plasmids compared to the parent polyethyleneimine polymers (70% for cyclodextrin‐linear polyethylenimine vs. 30% for bPEI and 75% cyclodextrin polyethylenimine vs. 40% for polyethylenimine). An important observation in this regard is that the cyclodextrin‐linear polyethyleneimine particles with PEGylation offer a better transfection in the curtailment of chloroquine. After IV administration into the tail vein of mice, this preparation ascertained fewer toxic effects, improved transfection efficiencies, and greater stability in the physiological salt solutions.

#### 5.2.5 3D bioengineered scaffolds and hydrogels

**FIGURE 9:**
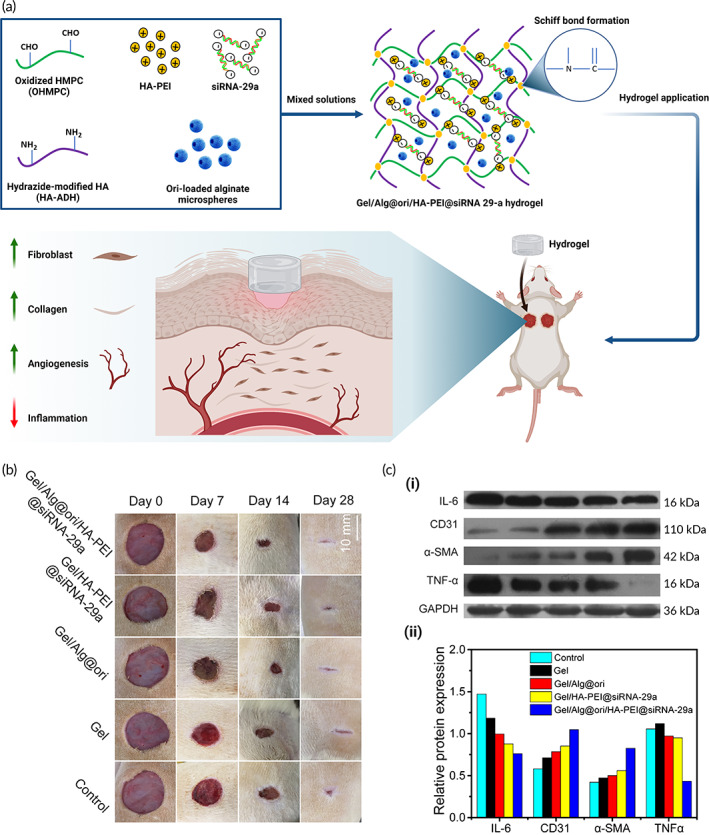
Schematic representation showing the preparation of Gel/Alg@ori/HA‐PEI@siRNA‐29a hydrogel and how siRNA‐29a loaded hydrogels accelerate the wound repair process. (a) Schematic representation of siRNA‐29a loaded hydrogels in wound healing and repair. (b) Digital photographs showing the process of healing on 0, 7, 10, 14, 21, and 24 days. (c) (i) Expression levels of α‐SMA, CD31, IL‐6, and TNF‐α using western blot techniques. (ii) Semiquantitative expression levels α‐SMA, CD31, IL‐6, and TNF‐α on day 7. Alg, alginate; Gel, Gelatin; HA, hyaluronic acid; Ori, oridonin; PEI, polyethyleneimine. Parts (b) and (c) are reprinted from Reference 225 with permission from Elsevier

Over the past few years, several advancements have been achieved in scaffolds and hydrogel‐based dressings to tackle the issues related to chronic wounds. For proper cell growth, proliferation, and differentiation at the wounded site, these novel 3D scaffolds and hydrogel‐based dressing provide a beneficial micro‐requisite environment around the wounded site. Hydrogels have been universally used in tissue engineering for the local release of growth factors,^221^ drug delivery applications,^222^ and as a 3D recyclable material for controlled cell growth and soft tissue regeneration.^223^ Recently, hydrogels have also found application in miRNA delivery for chronic wound management.^224^ In this work, a new hyaluronic acid and oxidized hydroxymethyl propyl cellulose loaded with siRNA‐29a hydrogel has been designed and integrated with oridonin micro/nanostructures, cross‐linked via Schiff base bonds (Figure 9).^225^

The synthesized nanointegrated hydrogels showed reasonable mechanical properties, excellent swelling, remarkable biodegradation, enhanced stability, and controlled release of oridonin and siRNA‐29a, satisfying basic conditions required by a modern dressing for accelerated wound healing. The overall in vivo experimental studies showed significantly enhanced diabetic wound healing activity, accelerated production of α‐SMA and CD31 angiogenic factors, and significant inhibition of pro‐inflammatory cytokines, that is, IL‐6 and TNF‐α has been found.^224^

**FIGURE 10:**
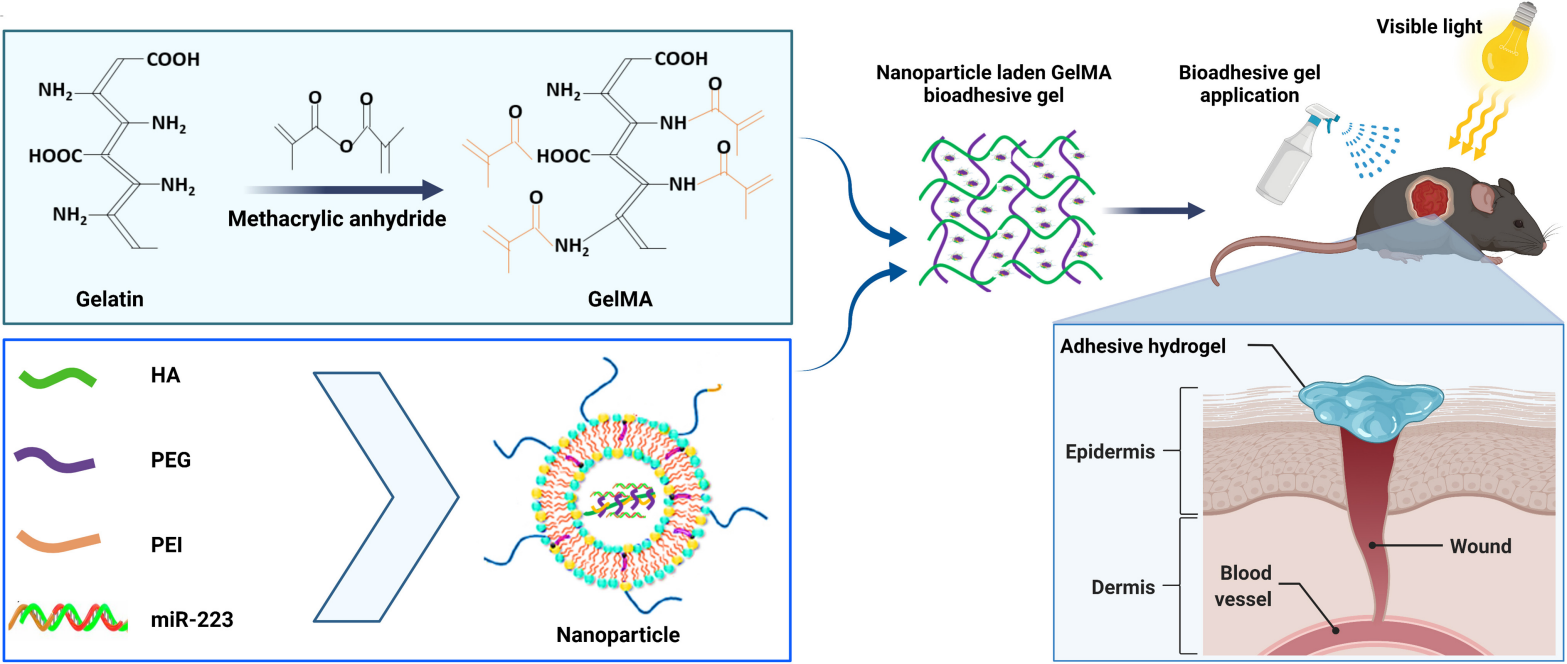
Schematic representation NP/miR‐223‐loaded gelatin methacryloyl hydrogels. Schematic illustration of the process involved in the synthesis of NA/miR‐233 loaded GelMA hydrogels and their application in wound healing

Local delivery of nucleic acid using a combinational approach of hydrogel dressing and nanocarriers helps in self‐administration by the patients, bypassing issues related to gastrointestinal tract absorption and first‐pass hepatic effect. Consequently, the use of targeted delivery helps improve the bioavailability and keep the concentration of therapeutic agents within the required therapeutic window. Above and beyond, localized delivery allows the maximum substantial portion of drug particles to the targeted site, maximizes the therapeutic efficacy, and reduced systemic drug toxicity. Although polymeric hydrogel dressings have been widely used to deliver numerous therapeutic molecules, the effective delivery of miRNAs via bioadhesives hydrogels for macrophage polarization with a technical challenge due to its negative charge, rapid loss by RNases, and small plasma half‐life of about 10 min.^158^ Therefore, a study conducted not long ago displayed the development of a gelatin meth acryloyl‐based bioadhesive hydrogel for localized delivery of miR‐223 5p mimic (miR‐223) encapsulated in hyaluronic acid NPs (Figure 10) for wound healing applications.^183^

To enhance the systemic circulation, two types of conjugation processes (HA‐polyethyleneimine [HA‐PEI] and HA‐polyethylene glycol [HA‐PEG]) have been employed to encapsulate miR‐223 based on their electrostatic interactions.^226^ They have used the HA‐PEG technique to increase residence time because of its anionic nature, presence in extracellular matrix, and positive involvement in various tissue regeneration and wound healing stages.^227^ The comprehensive study revealed the potential role in the polarization of macrophages to the wounded site and accelerated the wound healing process during in vivo studies.

## 6 CHALLENGES IN TRANSLATIONAL MEDICINE

The naked miRNAs are susceptible to degradation by endonucleases,^228^ have poor cellular uptake due to surface negative charge,^229^ face entrapment issues in endosomes,^230^ have low binding efficacy,^231^ and sometimes undergo off‐target delivery. Inadequate stability and consistency of the released miRNA molecule after local application is observed in blood circulation. However, degradation and endosomal escape are the most challenging task and hence several strategies have been developed such as using pH‐sensitive liposomes,^230^ cationic liposomes/NPs,^228^ light‐sensitive molecules,^232^ and bioengineered polymeric scaffolds and hydrogels.^233^

For proper cell proliferation, and cell differentiation at the wound site, the polymeric hydrogels and scaffolds provide an appropriate 3D micro‐requisite environment for local tissue repair and regeneration for controlled release of miRNA, thereby producing a suitable set of stimuli.^234^ Atelocollagen® is a type of collagen gel employed to treat skin disorders and other cosmetic surgeries.^233^ Atelocollagen® itself has been proposed for the controlled delivery of siRNA as well as miRNA. Moreover, Atelocollagen® is more effective for in vivo gene delivery. When it is confounded along with siRNA or miRNA, it is impervious to serum RNase nucleases and can be further proficiently transduced into cells.^235^ However, such hurdles are being presented in numerous ways to deliver potentially therapeutic miRNA modality using diverse carriers.^236^ For example, the quick degradation of the bare miRNAs by A‐type nucleases can be controlled with the help of chemical alterations, whereas their water‐soluble properties, higher molecular weight, and negative charge which chunk their permeation into the cellular membrane can be conquered by using different nanotechnology‐based delivery approaches.^237^ Apart from this, due to their favorable delivery and transport properties, nanoformulations and biomaterial have been reported to enhance the in vivo targeting, endow the formulations with the controlled release property, thus restricting their side effects and improving therapeutic outcomes.^238^, ^239^

The key stimulating constraint in miRNA delivery using nanotechnology is its low entrapment efficiency.^240^ Due to high‐water affinity, they undergo rapid diffusion into the aqueous phase when nanoprecipitation or emulsion‐based approach is being used, thus leading to decreased entrapment efficiency.^240^ Interestingly, NPs enhance the tissue and site‐specific distribution of miRNA, but the extent of enhancement is usually not sufficient. To date, numerous studies have been directed to fabricate proper surface functionalization of the nanomaterials with a specific ligand to realize receptor‐mediated endocytosis, thus dropping the necessary dose and adverse effects associated with the use of delivery systems.^241^

Apart from this, nanomaterial's colloidal stability in the biological milieu is useful for targeting miRNA delivery to a particular cell or tissue.^188^ Ideally, after administration, NPs should circulate in the blood flow until they get delivered to the targeted site. In addition, they should be premeditated in such a way that they undergo endosomal escape for appropriate interaction between the miRNA and its targeted cell/tissues.^242^ Numerous studies reveal that highly charged particles are predisposed to opsonization, especially positively charged, compared with neutral particles.^243^ Nevertheless, surface functionalization using polyethylene glycol or other surface modification approaches leads to an increased half‐life of the miRNA.

Notably, the type of disease decisively defines the physical and biological barriers that NPs should dominate the principal obstacles that are earlier mentioned associated with the miRNA itself and its delivery.^237^ Despite all the resources put into understanding miRNA technology and developing advanced delivery tools as well as expanding its applications, it has taken longer than expected to translate these results into clinical progress, thus limiting the clinical gain from these investments.^244^ Unconvincing hypotheses, inadequacies in preclinical models, statistical inaccuracies, and a lack of therapeutic relevance are the primary reasons for miRNA's challenges in translational medicine. To date, many miRNA‐based therapeutics have been used in clinical trials (https://clinicaltrials.gov/ct2/home) for numerous diseased conditions. But miR‐210 gene mediated therapy for chronic ulcer (ClinicalTrials.gov Identifier: NCT02024243), will be the world's first miRNA drug candidate, which will start its clinical trial by October 2022 and estimated date of completion is December 2023.

Additionally, in most of the cases, the inorganic NPs are taken up by the reticuloendothelial system shortly post‐administration hindering the delivery of miRNA to the wounded site and causing their nonspecific delivery to the unwounded sites. Although this issue might be addressed by surface PEGylation of the NP, the frequent use of PEGylated NP comes with a bundle of adversities including immunogenicity, and inadequate cell uptake. Furthermore, renal clearance is also considered to be one of the barriers to clinical translation. Furthermore, the industrial production of the delivery vectors imposes another challenge in their clinical applications. To translate inorganic nanomaterials into clinical practice, a method must be devised which makes NPs reproducible from batch to batch. Because of the complex physicochemical and structural characteristics of NPs themselves, nanoplatforms that require complex synthetic procedures pose a serious hindrance to large‐scale pharmaceutical production, making the clinical translation of NPs very difficult.^245^ Moreover, in the case of viral vectors, the absence of standard analytical techniques for comparing titer and product evaluation acts as a challenge. Insufficient resources are believed to be the cause of this problem. Additionally, process development for viral vectors does not include integrated continuous bioprocessing strategies. In spite of the commercial availability of small‐scale STR platforms and equipment (e.g., DASbox® and ambr15®), little is known about their application as viral vector development platforms.^246^

Another major challenge in the clinical translation of nanotechnology‐based vehicles for miRNA delivery involves their associated toxicity development due to the accumulated nanomaterial in the nonspecific sites. When the nanocompounds enter the body, they penetrate through the cell boundaries and damages mitochondria, causing a mutation in DNA and ultimately resulting in cell death.^247^ Moreover, the underlying mechanism for nanomedicine toxicity also involves the release of reactive oxygen species which might lead to oxidative stress and consecutive inflammatory processes thus affecting protein synthesis, cell membrane, and DNA.^248^ For example, toxicity due to gold NPs may be attributed to their physicochemical characteristics leading to the production of reactive oxygen species and oxidative stress. Their shape is considered a factor behind the toxic effect that is gold nanostars are highly toxic compared to gold nanospheres.^249^ Silver NPs also show toxicity in tissues at high doses due to the nonspecific release of silver ions in the healthy tissues.^250^ Regulatory issues are yet another concern regarding the gap between laboratory research and clinical use. Inorganic nanomaterials are frequently denied regulatory approval despite positive clinical results owing to concerns from the government, including inappropriate design of endpoints, inadequate justification on clinical comparator selection, and insufficient analysis methodology for clinical data. Thus, pharmaceutical companies must solicit feedback from regulatory agencies during the whole nanomaterial development process so as to prevent delay or denial of regulatory approval.^245^

## 7 CONCLUSION AND FUTURE PERSPECTIVES

During the past decades, a tremendous amount of research and clinical trials have been performed on the identification and development of biological factors that modulate the physiological processes throughout wound healing to enhance the healing rate and quality. However, the growing population of patients suffering from chronic wounds or scarring demonstrates the need for more efficient strategies in wound healing. miRNAs present attractive therapeutic candidates to develop safe, simple, and effective strategies, capable of modulating various biological mechanisms for improved wound healing. However, due to their limited stability and membrane permeability, the main limitation of miRNA‐based therapy is their inefficient delivery to the target site. Therefore, future investigations need to develop novel delivery systems for the efficient delivery of miRNAs without an adverse immune response.

To date, multiple drug carriers have been identified and developed for the successful delivery of miRNAs such as liposomes, cationic polymeric NPs, scaffolds, hydrogels, and even inorganic materials. However, in addition to the drug carrier, the delivery strategy that controls the spatiotemporal release profile is a very important factor. However, less attention has been paid to the proper delivery tool. An optimal drug delivery system should release the specific therapeutic agents corresponding to the spatiotemporal requirements throughout wound healing stages.^251^ Various drug delivery systems, which can control the temporal release profile, including passive, active, and smart systems can be used for miRNA‐based wound therapy.^11^ Smart systems can either incorporate smart materials reacting to wound biomarkers, or integrated sensing/delivery systems.

Furthermore, to control spatial distribution of the miRNA in the wound bed, two main approaches including topical and intradermal/transdermal delivery can be implemented.^7^ While most investigations have been performed on the application of topical systems, in which the drug with its carrier is administrated directly on top of the wound, there is an emerging interest toward the intradermal delivery systems, in which the drug is delivered into the deeper layers of the wound bed.^3^ The intradermal methods provide the opportunity to enhance the local availability of the drug in the wound bed through bypassing the eschar and bottom‐up exudate flow, rich with various enzymes, which can disrupt the integrity of the miRNAs, even when delivered with drug carriers. Therefore, a more efficient miRNA delivery is expected using intradermal systems for future investigations.

Additionally, miRNA delivery can be combined with current wound management strategies including tissue debridement, infection treatment, moisture control, and frequent monitoring of the wound edge advancement. Hence, it is predicted that miRNA can become a new generation of nucleic acid therapies for chronic wounds management using efficient delivery approaches.

Future investigations should further highlight the influence of various signals in the wound microenvironment on miRNA expression and function. In addition, identification of the furthermost appropriate transporter for miRNA delivery and the exact targets of miRNAs in each tissue type signifies the subsequent milestones regarding miRNA‐based therapy approaches. Overall, as a concluding statement of this article, the authors highlight how miRNAs modulate cellular behavior and subsequent tissue regeneration. Due to the complicated and multidisciplinary nature of miRNA‐based wound healing, close and synergistic teamwork among experts from diverse areas including bioengineering, biomedical science, nanotechnology, and biotechnology is of great importance to translate this technology into clinical applications.

## AUTHOR CONTRIBUTIONS

**Asmita Deka Dey:** Writing – original draft (equal). **Satar Yousefiasl:** Writing – original draft (equal); writing review and editing (equal); figures preparation. **Arun Kumar:** Writing – original draft (equal). **Farnaz Dabbagh Moghaddam:** Writing – review and editing section 2 (equal); preparing figure 2. **Ilnaz Rahimmanesh:** Writing – original draft (equal); preparation figures 5 and 6. **Mohamadmahdi Samandari:** Writing – review and editing (supporting). **Sumit Jamwal:** Writing – original draft (equal). **Aziz Maleki:** writing – review and editing (supporting). **Abbas Mohammadi:** writing – review and editing (supporting). **Ana Cláudia Paiva‐Santos:** Writing – review and editing (supporting). **Ali Tamayol:** Writing – review and editing (supporting). **Esmaeel sharifi**: Writing – review and editing, and commenting on the draft (Lead). **Pooyan makvandi**: Writing – review and editing, and commenting on the draft (Lead). 

## CONFLICT OF INTEREST

The authors declare no potential conflicts of interest.

## Data Availability

Not available.
